# Genome-wide Membrane Protein Structure Prediction

**DOI:** 10.2174/13892029113149990009

**Published:** 2013-08

**Authors:** Stefano Piccoli, Eda Suku, Marianna Garonzi, Alejandro Giorgetti

**Affiliations:** 1 Applied Bioinformatics Group, Dept. of Biotechnology, University of Verona, strada Le grazie 15, 37134, Verona, Italy;; 2 German Research School for Simulation Sciences, Juelich, Germany;; 3 Center for Biomedical Computing (CBMC), University of Verona, strada Le grazie 8, 37134, Verona, Italy

**Keywords:** Genome-wide scale analysis, Homology modeling, Human membrane proteome, Multitasking approach, Protein structural bioinformatics, Membrane protein.

## Abstract

Transmembrane proteins allow cells to extensively communicate with the external world in a very accurate and specific way. They form principal nodes in several signaling pathways and attract large interest in therapeutic intervention, as the majority pharmaceutical compounds target membrane proteins. Thus, according to the current genome annotation methods, a detailed structural/functional characterization at the protein level of each of the elements codified in the genome is also required. The extreme difficulty in obtaining high-resolution three-dimensional structures, calls for computational approaches. Here we review to which extent the efforts made in the last few years, combining the structural characterization of membrane proteins with protein bioinformatics techniques, could help describing membrane proteins at a genome-wide scale. In particular we analyze the use of comparative modeling techniques as a way of overcoming the lack of high-resolution three-dimensional structures in the human membrane proteome.

## INTRODUCTION

A lipidic membrane sorrounds cells with the aim of isolating them from the external world. This barrier has also an important role in cellular communication because it is the target of all the extracellular stimuli acting on the cell. So the membrane presents a major target for environmental stimuli acting upon a living cell. Several proteins, i.e. integral membrane proteins, are thus specialized in detecting extra cellular signals and translating the information to the cell, allowing a response. Membrane proteins are involved in several signaling pathways. 

The original human genome sequencing project estimated 20% of the total gene count of 31,778 genes to code for membrane proteins [1]. This enormous number indicates the importance of membrane proteins for organism survival. Unfortunately, due to the difficulties in expression at the experimental level, just few of them have been deeply characterized. For example, if we consider the total amount of membrane proteins for which the three-dimensional structure is known, a feature absolutely needed for a complete functional characterization, we can observe that the structure of just 397 unique membrane proteins has been solved by X-ray crystallography (http://blanco.biomol.uci.edu/mpstruc/listAll/list). A total of 40 of them are human membrane proteins (38 transmembrane alpha protein and 2 monotopic membrane protein). While 26 of these structures were solved by X-ray crystallography just 14 were solved by Nuclear magnetic resonance (NMR) techniques. These numbers represent a small portion of the entire human membrane proteome. It is evident from the structures that have been deposited so far, that only all-alpha and beta-barrel structural organizations are present in nature. Indeed most of membrane proteins in the Protein Data Bank (PDB: http://www.pdb.org), i.e. 67%, consist of bundles of transmembrane helices with different tilting with respect to the membrane plane and to each other. 

Recently, membrane protein crystallography techniques have reached higher levels of applicability, allowing the resolution of proteins of the upmost importance for the scientific community. Anyway, although the revolution in ‘resolution’ techniques, we have to admit that there is still an enormous gap between annotated protein sequences and structures. The need of membrane protein characterization, thus calls for alternative innovative approaches. One of these consisted in using an extensive combination of computational biology techniques with molecular biology validating experiments. 

Here we review how these could help describing membrane proteins at a genome-wide scale. In particular, we will try to give a numerical idea about the possibility to use homology modeling techniques in order to obtain structural models of all the human membrane proteome. 

## PROTEIN STRUCTURAL BIOINFORMATICS

**Fig. (1):**
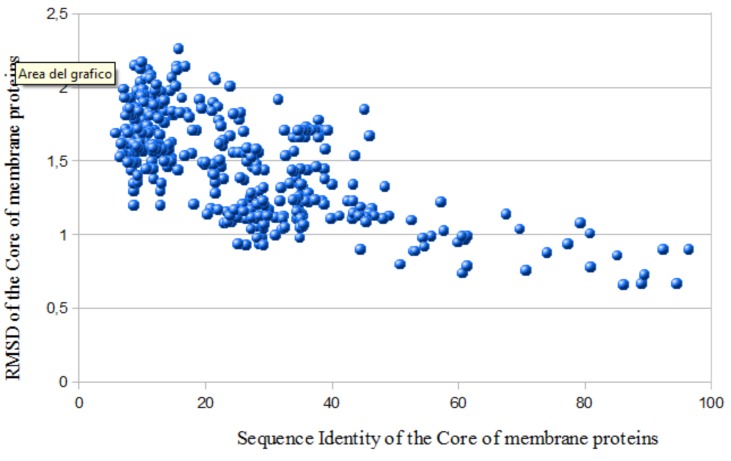
RMSD versus % Sequence Identity of the core of membrane proteins. The core of all the membrane proteins found in the Membrane Proteins with Known Structure Database (http://blanco.biomol.uci.edu/mpstruc/listAll/list) was aligned at the structural and sequence level. The relationship between structural similarity (D) and the fraction of mutated residues (H) calculated by performing a least square fit of the data is: D=0,71E^0,93H^ (black continuous curve).

Proteins are linear polymers of aminoacidic residues, modeled by events of random change and natural selection along a period of millions of years, fulfilling a delicate sequence-structure-function relationship. Thus, evolutionarily related proteins (homologs) that descend from a common ancestor accumulated small changes still conserving the same general fold. This forms the basis of the technique known as comparative or homology modeling [2, 3]. Chothia and Lesk, in a seminal article [4] have aligned the sequences and structures of all the soluble proteins with known structure (up to 1986), finding a correlation between the evolutionary distance and structural divergence between evolutionary related proteins. Summarizing, comparative modeling is a method that allows the prediction of protein structures using as a template a member of the family for which the three-dimensional structure (3D-structure) is known [2, 3]. Very recently, we have checked for the existence of correlation between evolutionary relationship and structure similarity on membrane proteins, following the same protocol as Chothia and Lesk [4, 5]. By using the LGA server (http://proteinmodel.org/) we have aligned, at the sequence and structural level, the core region of all the membrane proteins with known three-dimensional structure (565 pairs) and produced the graph of (Fig. **1**) (Here we have improved the graph by including the recently solved protein structures). The structural divergence between two evolutionary correlated proteins is measured as their Root Mean Square Deviation (RMSD).

For comparison purposes the core of the protein was considered as composed by all the amino acids present in secondary structure elements and those regions not diverging for more than 3 Å, as in [4]. The p value was 2,2E^-73^, indicating the statistical significance of the graph. We have then calculated the least square fit of the data, as performed in [4], obtaining the black continuous curve of (Fig. **1**). The calculated R^2 ^=0.83 value demonstrates the high correlation between structure and sequence similarity. If we consider that Chothia and Lesk have calculated that plot more than 28 years ago considering just 32 pairs of soluble proteins, we have to admit that the result for 565 pairs of membrane proteins is astonishing. Although the noise in the low identity region (high fraction of mutated residues) seems to be higher, in the last few years (see Application Cases), several membrane proteins, present in this ‘twilight region’, that is, sharing sequence identities lower than 20% with their templates, were successfully modeled.

The question at this point can be: Would it be possible to obtain accurate structural models of the entire human membrane proteome?

## TRANSMEMBRANE DOMAIN MODELING STATISTICS

A recent project, *Survey of the human transmembrane proteome* [6], allowed the assessment of the feasibility for predicting the structure by comparative modeling techniques of the human membrane protein domains. The tools and the database developed in the ambit of this project are associated with ModPipe [7], an automated modelling pipeline. In this project, all the human transmembrane all-α domains were identified, clustered and classified into three different classes, i.e. enzymes, transporters and receptors, totalizing 3417 non redundant domains with at least two α -helices. These domains were then funneled through two different automatic pipelines, i.e. ModPipe and BLASTCLUST [8], with the aim of identifying putative templates. While with ModPipe templates were identified for 984 domains (threshold level of 25% sequence identity - SI), BLASTCLUST found 1201 ones (threshold level of 25% SI and 70% of coverage). We have repeated the procedure for searching homologues of known structure of the human transmembrane domains using an additional methodology based on the program HMMER [9] and considering e-value thresholds instead of SI. Each of the 3838 domain was used for the generation of Hidden Markov profiles, (https://modbase. compbio.ucsf.edu/projects/membrane/downloads: Domain Sequences THM). The HMMER program was then used to align the profiles against all the proteins archived in the Protein Data Bank (PDB: http://www.pdb.org). We found at least one template for 1881 domains with the e-value lower than the threshold (<0.01). The analysis of the coverage of the 1881 sequences led to the following results: 215 domains with 100% coverage, 979 with a coverage greater than 70%, 276 with a coverage between 50% and 70% and 420 domains with a coverage between 50% and 20%. These numbers, obtained by different techniques, show that around 1/3 of the human membrane proteome could be modeled using comparative modeling techniques with different levels of accuracy. 

## MEMBRANE PROTEIN MODELING: APPLICATION CASES

In order to assess the use of homology modeling techniques in membrane protein characterization we analyzed the literature of the last 5 years. From NCBI-Pubmed we found 408 research articles and 33 reviews. We have analyzed the contributions considering the three main categories of membrane proteins, i.e. transport proteins, receptors and membrane enzymes [1]. For each category we selected representative articles in which homology modeling gives a decisive contribution. 

### Transporters

Homology modeling technique was used for example, i) to study the conformational changes between the holo and apo physiological states of the ATP-binding cassette (ABC) superfamily of proteins [10, 11], ii) to study the water and glicerol permeability and response to drug inhibitors of aquaporins [12, 13]. The latter was an example in which the combination of computational techniques with experiments, i.e. stopped-flow analysis and inhibitions assays, allowed the description of complex systems. In order to study Cys-loop ligand-gated ion channels like g-amino butyric acid type A receptors (GABAARs) and glycine receptors (GlyRs), modeling data were used to set up mutagenesis experiments aimed at characterizing glycosilation sites found to be altered in disease states of neuronal activity [14-16]. Voltage-gated proton channels (HV1s) homology models combined with electrophysiology experiments, like patch clamp, were used to characterize the open conformation and accessibilities of the channel [17]. Cation channels (Na+, K+, Ca2+) modeling sessions were instead used to characterize the different activation states of the human ether-a-go-go related gene 1 (hERG1) K+ ion channel [18] and of the CaV1.2 calcium channels [19]. The activation mechanisms in cyclic nucleotide channels have been also characterized by the use of a combination of site-directed mutagenesis experiments and homology modeling techniques [20-23]. Using molecular dynamic and metadynamics (MTD), a method that allows exploring multidimensional free energy surfaces (FESs), an alternative Na+ binding site of Sodium-Galactose Transporter (SGLT) symporter protein was predicted [24]. Homology Modeling combined with virtual docking simulations was used to study the interaction between Acid-sensing ASIC channels and toxins [25]. Kranjc [26] applied homology modeling in order to construct a model of arginine rich domain of the calcium-activated anion channel bestrophin and evaluate how specific mutations affect its capacity to bind calcium ions. The works presented in this section represent the power of combined experimental/computational approaches. Indeed, from what regards ion channels, they gave a fundamental contribution to the understanding of the molecular determinants of the gating mechanisms and helped to characterize the role of residues involved in ion binding at atomic level. In the case of carrier and solute transporters the computational/experimental characterization of the transport mechanisms allowed the gain insights into the large conformational changes that occur upon transportation of solutes.

For recent and extensive reviews on this category see [27-29].

### Receptors

Homology modeling was also extensively used in the last years to unravel the functioning and structural features of given transmembrane receptors. For example TLR8, a member of the Toll-like receptors (TLRs) family, were studied by a combination of comparative modeling, molecular dynamics simulations and virtual docking and computational mutagenesis studies. These studies were carried out in order to investigate the interactions of the receptors and its cognate antiviral compound R848 that activates the full TLR8 pathway [30]. Scavenger receptors, expressed by endothelial cells (SREC-I), are membrane protein involved in the endocytosis of lipoproteins: trough homology modeling and experimental procedures the effect of glycosilation on its physiological activities was studied [31]. 

G protein-coupled receptors (GPCRs) form the largest membrane-bound receptor family expressed by mammalians (encompassing ca. 4% of the protein-coding human genome, 25) and are of paramount importance for pharmaceutical intervention (ca. 40% of currently marketed drugs target GPCRs) [32]. Recently, there was an explosion in the crystallography of GPCRs [33-37] determining a breakthrough in the field of signaling processes. It was of the outmost importance for the scientific community, indeed, the 2012 Nobel Prize in Chemistry was awarded to Brian Kobilka (Stanford University) and Robert Lefkowitz (Duke University) for their structural work on the GPCRs. Anyway, while about 5500 GPCR sequences are public, there are actually only 19 crystallized structures (as reported in the http://blanco.biomol.uci.edu/mpstruc/listAll/list) providing exciting opportu- nities for structure-based drug design methods that can now use increasingly reliable homology models of GPCR targets [38, 39]. Successful computational models of GPCRs have been used for virtual screening, enriching the rate of ligand hits relative to a random collection of compounds, with hit rates ranging from 3 to 21% [40, 41], comparable to virtual screening success rates with X-ray structures [38]. Furthermore, research aimed at elucidating the underlying principles determining the molecular responsiveness range of GPCRs that mediate senses, such as taste [42, 43] and odor [44] receptors, depends on the ability to build reliable models of the interaction sites. A crucial step in understanding specificity and promiscuity in molecular recognition and structure-based design is to identify the residues that are important for ligand binding. Researchers have successfully applied homology-based of non-rhodopsin GPCRs structure modeling approaches to ligand-binding elucidation [41, 45, 46]. Interestingly, the bigger amount of works based on homology modeling (we found 88 articles by last five years) is dedicated to the rhodopsin-like GPCRs (class A), [47-49] and Taste2 (T2R) GPCRs [43, 50-53]. Several approaches to modeling GPCRs have been described [41], including *ab initio* [42, 54] and template based [40, 49, 55]. The relevance of the articles presented here resides in the fact that the combination of homology modeling and/or molecular docking combined with a rapid growing number of protein structures deposited in the PDB database, together with the availability of functional assays, allowed to represent the ligand-binding interactions at an unprecedented level of detail. This kind of approaches would allow in a near future the intelligent design of pharmaceutical compounds, using a structure-based strategy. For an extensive review on GPCRs see [56]. 

Recently Malo *et al.* [57] proposed a new method to generate all-atom models of the membrane-spanning part of TM proteins that repacks secondary structure elements of a homology model guided by a ligand and a limited set of experimental and evolutionary restraints. 

### Membrane Enzymes

Homology modeling techniques were also applied to the characterization of membrane enzymes. Using molecular modeling validated by site-directed mutagenesis experiments, the molecular interaction between F1-ATPase and the glycine-extended form of gastrin G-gly was characterized [58]. The active site residues of the 25-hydroxycholesterol-7α-hydroxylase and oxysterol-7α-hydroxylase CYP7B1, CYP7 family members were characterized by comparative modeling techniques combined with virtual docking experiments and validated using site-directed mutational analysis [59]. These works were the first attempts in which a systematic experimental/computational combined efforts were applied to this family of enzymes, and allowed the identification of fundamental residues involved in ligand/substrate binding.

## METHODOLOGIES FOR GENOME-WIDE MODELING APPROACHES

The possibility of obtaining high quality models through the use of comparative modeling techniques will allow the development of automatic tools able to perform genome-wide membrane protein modeling. Indeed, in the last few years several efforts in this direction have been carried out for proteins in general, using two methods: ModPipe [7] and the Modelling and Assessment of ISoforms Through Automated Server (MAISTAS) [60], a tool particularly designed for modelling all the annotated isoform(s) of a protein sequence. For the specific case of membrane proteins, some of us have recently developed a server dedicated to the automated modelling of GPCRs, i.e. the GPCRs Online Modelling and Docking webserver (GOMoDo) (http://molsim.sci. univr.it/gomodo). This web tool performs automatic homology modelling and ligand docking of GPCR receptors. Moreover, in the next future it will be extended to offer the user the possibility of modelling membrane proteins in an automatized way, contributing to the automatic modelling of the entire human membrane proteome. 

In the case of the 2/3 of proteins that could not be covered by homology modelling, efforts have been also undertaken by implementing techniques such as fold recognition, threading in particular. Indeed, in the ambit of the Encode project, recently Jones and collaborators have used the GenTHREADER [61, 62] method to automatically model the structure of a set of protein variants that could not be modelled by homology modelling techniques [63]. This approach could be extended to the human membrane proteome, until the Structural genomics projects will be able to offer at least a template for each of the families of membrane proteins. Another server is the Bologna Annotation Resource Plus (BAR+) [64] that is based on a large-scale genome cross comparison, taking advantage of a huge pairwise sequence comparison that includes 988 complete proteomes. One of the principal features, is the structure prediction at genome-wide level, using fold recognition methods. 

## CONCLUSION

Membrane proteins are of the outmost importance for the survival of any living being. A deep insight into the molecular mechanisms underlying their function is thus needed for a complete characterization of the way our cells exchange information with the environment. A detailed annotation of the human membrane proteome implies necessarily, the structural characterization of membrane proteins. As stated in the previous sections, structural biologists still find difficult to overcome the implicit limitations of crystallographic techniques on membrane proteins. These difficulties call for techniques that combine *in vitro* and *in silico* approaches. 

Here we have reviewed novel biological applications in which combinations of experimental/computational multitasking approaches were shown to be of fundamental importance for obtaining a deep characterization of complex systems that include membrane proteins. In particular, homology modeling represents a powerful instrument because it is applicable to many different biological systems. Indeed, the astonishing improvements in crystallographic techniques combined with comparative modeling techniques will allow the characterization of almost the complete human membrane proteome in the near future.
